# Learnings from Regional Market Dynamics of Originator and Biosimilar Infliximab and Etanercept in Germany

**DOI:** 10.3390/ph13100324

**Published:** 2020-10-21

**Authors:** Evelien Moorkens, Teresa Barcina Lacosta, Arnold G. Vulto, Martin Schulz, Gabriele Gradl, Salka Enners, Gisbert Selke, Isabelle Huys, Steven Simoens

**Affiliations:** 1 Department of Pharmaceutical and Pharmacological Sciences, KU Leuven, 3000 Leuven, Belgium; evelien.moorkens@kuleuven.be (E.M.); teresa.barcina@kuleuven.be (T.B.L.); isabelle.huys@kuleuven.be (I.H.); steven.simoens@kuleuven.be (S.S.); 2 Hospital Pharmacy, Erasmus University Medical Center, 3015 Rotterdam, The Netherlands; 3 Institute of Pharmacy, Freie Universität Berlin, 14195 Berlin, Germany; 4 Department of Medicine, ABDA—Federal Union of German Associations of Pharmacists, 10557 Berlin, Germany; 5 Deutsches Arzneiprüfungsinstitut e. V. (DAPI), 10557 Berlin, Germany; m.schulz@dapi.de (M.S.); G.Gradl@dapi.de (G.G.); S.Enners@dapi.de (S.E.); 6 AOK Research Institute (WIdO), 10178 Berlin, Germany; gisbert.selke@wido.bv.aok.de

**Keywords:** infliximab, etanercept, TNFα inhibitors, biologics, biosimilars, Germany, policies, incentives, uptake, market dynamics

## Abstract

Drug budget and prescription control measures are implemented regionally in Germany, meaning that the uptake of pharmaceuticals, including biosimilars, can vary by region. We examine regional market dynamics of tumor necrosis factor alpha (TNFα) inhibitor originators and biosimilars in Germany and studied the influence of biosimilar policies on these dynamics. This study is based on: (1) a literature review in which German biosimilar policies are identified, (2) the analysis of dispensing data (2010–2018) for the class of TNFα inhibitors, and (3) ten semi-structured interviews investigating prescribers’ and insurers’ views on factors potentially influencing biosimilar uptake. The analysis of biosimilar market shares of infliximab and etanercept revealed wide variations across the 17 German Regional Associations of Statutory Health Insurance Accredited Physicians (PA regions). Quantitative analyses indicated that biosimilar market shares for infliximab and etanercept were significantly lower in former East Germany when compared to former West Germany regions. Through qualitative interview analyses, this study showed that the use of infliximab and etanercept biosimilars across Germany is primarily influenced by (1) the regional-level implementation of biosimilar quotas and the presence of monitoring/sanctioning mechanisms to ensure adherence to these quotas, (2) the different insurer-manufacturer discount contracts, and (3) gainsharing arrangements established at the insurer-prescriber level.

## 1. Introduction

The incorporation of biologic therapies into clinical practice has positively transformed health outcomes for many patients diagnosed with severe and highly debilitating chronic conditions [1,2,3]. As a result, these therapies have represented a growing market in recent decades (accumulated global sales of USD 312 billion in 2018) [4]. Being approved for an increasing number of disease areas, high-cost biologic therapies constitute an important budget impact to be managed by healthcare systems across Europe [5]. However, with the expiration of patents and other exclusivity rights for biologics, non-innovator and therapeutically equivalent versions, i.e., biosimilars, enter the market with the potential to create competition within the therapeutic classes [6]. This leads to altered market dynamics and potentially to decreasing treatment costs and increasing patient access to biologic therapies [7].

Germany, with 83 million inhabitants [8], is the most populous country of the European Union and is an important market for biologics and biosimilars [9,10]. Here, sales of biologics amounted to EUR 11 billion in 2017 [11]. An important class of biologics that has been subject to competition by biosimilars is that of TNFα inhibitors (sales of EUR 2.2 billion in 2017 for Germany) [4]. Five active substances with a TNFα neutralizing activity (infliximab, etanercept, adalimumab, golimumab and certolizumab pegol) are available for the treatment of immune-mediated inflammatory diseases [12]. The originator products—Remicade^®^ (infliximab), Enbrel^®^ (etanercept) and Humira^®^ (adalimumab)—have been present in the German pharmaceuticals market for more than 15 years now. In 2013, infliximab biosimilars under the names Inflectra^®^ and Remsima^®^ received marketing authorization by the European Medicines Agency (EMA) and accessed different European markets. Consequently, in 2015, Inflectra^®^ and Remsima^®^ were launched in Germany. They were followed by the market entry of infliximab biosimilars Flixabi^®^ (2016) and Zessly^®^ (2018). In the case of etanercept, biosimilar products Benepali^®^ and Erelzi^®^ were brought to the German market in 2016 and 2017, respectively [11]. The offer of TNFα inhibitors has been expanded with the incorporation of adalimumab biosimilars Imraldi^®^, Hyrimoz^®^, Amgevita^®^, Hulio^®^ in the last quarter of 2018 and the posterior market launch of Idacio ^®^ (2019) and awaits further developments, once exclusivity rights for Cimzia^®^ (certolizumab pegol) and Simponi^®^ (golimumab) have expired in 2021 and 2024, respectively [13,14].

The German law for more safety in the supply of pharmaceuticals (German: Gesetz für mehr Sicherheit in der Arzneimittelversorgung, GSAV June 2019) has been recently amended to optimize the use of biosimilar products as a cost-containment tool [15]. The introduced changes would provide a more favorable environment for the close monitoring of biosimilar regional market dynamics at the federal level. This is of relevance based on the decentralized organization of the German healthcare system, where the German regions are responsible for managing prescription and drug budget control activities. Regional differences in biosimilar policies and practices have been associated with the heterogeneous uptake of biosimilars between product classes and across regions [15,16,17,18]. In Germany, differences in biosimilar market shares were described for TNFα inhibitors at the end of 2018: in Westphalia-Lippe and Lower Saxony, biosimilar uptake was two times higher than in Baden-Württemberg. However, reasons behind the variable uptake of biosimilars across the 17 German Regional Associations of Statutory Health Insurance Accredited Physicians (PA regions; German: Kassenärztliche Vereinigungen (KV)) have not been examined in detail [19].

In this study, we analyze the regional market dynamics of TNFα inhibitors following the entry of biosimilars for infliximab and etanercept, and investigate the influence of diverse factors, especially biosimilar policies and practices, on biosimilar uptake. This study builds on previous research analyzing regional market dynamics of infliximab and etanercept originators and biosimilars in Sweden (see Box 1) [17,18].

Box 1 **Summary of what is already known about this topic and the added value of the study.**

**What is already known about this topic**

- Regional variations in the use of TNF⍺ inhibitor biosimilars in Sweden have been attributed to the extent of actual (discounted/rebated) price differences between biosimilars and the originator product, the engagement of key opinion leaders, the issuance of local guidelines and to gainsharing arrangements [17,18].
- In Germany, biosimilar uptake is also known to vary at the regional level. This was investigated by Blankart et al. for erythropoiesis-stimulating substances, filgrastim and somatropin. Variations in biosimilar uptake were partly attributed to the presence of explicit regional cost-control measures, such as quota regulations [54].

**What this study adds**

- Although previous studies have characterized regional variations in the uptake of TNF⍺ inhibitor biosimilars in Germany, the reasons behind this variable uptake have not been examined in detail [19].
- This study highlights the influence of prescription and budget control activities (organized at the regional and insurer level) on the variable uptake of infliximab and etanercept biosimilars.

## 2. Results

### 2.1. Overview of TNFα Inhibitor Dynamics in the German Healthcare System

#### 2.1.1. The German Market for TNFα Inhibitors

In Germany, the federal and regional governments delegate certain healthcare responsibilities to self-regulated organizations of payers and providers that can operate within the Statutory Health Insurance (SHI; German: Gesetzliche Krankenversicherung (GKV)) scheme or the substitutive Private Health Insurance (PHI; German: Private Krankenversicherung (PKV)) scheme. Within SHI, the main payers are multiple membership-based not-for-profit insurance companies (sickness funds; German: Krankenkassen), which may function nationwide or at the regional level [20]. According to SHI data, originator products Humira^®^, Enbrel^®^ and Simponi^®^ and a biosimilar version of Enbrel^®^ (Benepali^®^) still ranked in 2018 among the top 30 contributors to pharmaceutical expenditure [19]. Due to the scheme for care delivery in Germany, most of this expenditure is managed by the ambulatory sector, through which the majority of prescriptions for TNFα inhibitors are issued. In Germany, there is a clear distinction in the provision of outpatient and hospital care. Outpatient care is delivered by individual doctor practices and specialized medical centers, where services are provided that are usually a hospital competence across Europe [21,22]. In this sense, most prescriptions for intravenous infliximab (70% issued by gastroenterologists) and subcutaneous etanercept (87% issued by rheumatologists) go through the German ambulatory sector [11]. Within this sector, sickness funds negotiate overall prescription budgets with the Federal Association of Statutory Health Insurance Physicians (German: Kassenärztliche Bundesvereinigung (KBV)). They also contract prescription budgets for the regions through negotiations with the 17 German PA regions. Although Germany is divided into 16 federal states (German: Bundesländer), the areas Northrhine and Westphalia-Lippe within the state of Northrhine-Westphalia are represented by two independent PA regions [19].

#### 2.1.2. Regulations of the German Market for TNFα Inhibitors

TNFα inhibitor therapies entered the German market under a free-pricing and full reimbursement scheme (up to a patient co-payment of at most EUR 10 per pack dispensed). However, lower list prices are expected for biosimilars when compared to the originator. While price setting for pharmaceuticals in the hospital market is unregulated and established through direct hospital-manufacturer negotiations, some instruments for regulation are applicable to the retail market [19]. For example, reference price groups were established for infliximab and etanercept in accordance with §35 German Social Code Book V (German: Sozialgesetzbuch V (SGB V)) [23]; the reference price acts as a reimbursement limit, with any overshooting cost borne by the patient. The inclusion of all infliximab-containing products into a reference price group resulted in a 22% reduction in Remicade^®^’s selling price at the end of 2018 [19]. Until recently, the German Legislation (§129 SBG V) only allowed automatic substitution for bioidenticals (i.e., biosimilars made by the same production site and process, as is the case for Inflectra^®^ and Remsima^®^) [19]. Modifications of the law for more safety in the supply of pharmaceuticals (GSAV) have extended these regulations to non-bioidentical biosimilars, provided that the Federal Joint Committee (German: Gemeinsamer Bundesausschuss (G-BA)) recognizes interchangeability [15]. Based on the restrictions for automatic substitution of biologics, the type of procurement contract (§130a SGB V) usually applied to generics has been deemed inadequate for biosimilars. Despite the various alternative procurement mechanisms possible (e.g., tendering), insurance companies mostly rely on the organization of “open-house rebate contracts” (German: Open-House-Rabattverträge) in which all suppliers of originator biologics and biosimilars can participate. Participants in “open-house rebate contracts” qualify to sign a supply contract if they adhere to certain pre-defined contractual conditions, including mandatory discounts on list prices. These conditions are freely set by the insurer and cannot depend on individual negotiations with certain suppliers [19].

The market for TNFα inhibitors is indirectly regulated through the establishment of prescribing targets. Every year, the National Association of Statutory Health Insurance Funds (German: Gesetzliche Krankenversicherung-Spitzenverband (GKV-SV) and KBV agree on target areas for prescribing control (biosimilars included). For these areas, they use the previous year’s prescription rates to set recommendations. The output of this negotiation is reflected in a non-binding contract (German: Bundesrahmenvorgaben für die Arzneimittelvereinbarungen) that serves as a guideline for regional agreements. Insurer companies and regional physician associations look at the national advisory agreement and define implementation details for contracts which are binding at the regional level (German: Arzneimittelvereinbarungen). Minor deviations are allowed, as long as the overall cost-containment effect is achieved. This means that regional physician associations are not forced to rely on biosimilar prescription quotas. Instead, they can give more importance to alternative cost-containment mechanisms that still meet the general objective [19].

### 2.2. Analysis of Dispensing Data for TNFα Inhibitors

#### 2.2.1. TNFα Inhibitor Products: Evolution in Sales Volume

**Figure 1:**
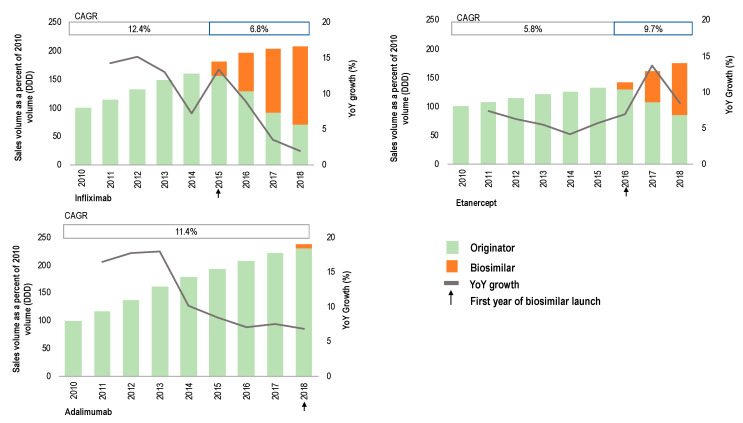
Sales volume evolution (2010–2018) expressed as a percentage of 2010 volume and measured as defined daily doses (DDDs) for originator and biosimilar products containing infliximab, etanercept and adalimumab (primary axis). Year-over-year (YoY) growth (%) is represented on the secondary axis. Compound annual growth rate (CAGR) is calculated before and after biosimilar launch. The graphical representation of the data is based on a figure published by IQVIA [26].

Overall, in Germany, the sales volume of TNFα inhibitor products has increased over time (from 17.68M defined daily doses (DDDs) in 2010 to 42.06M DDDs in 2018). From 2010 to 2018, sales volume for infliximab and adalimumab increased over two-fold, and 1.8-fold for etanercept (see Figure 1). The rise in the sales volume of TNFα inhibitors has been attributed to several factors (e.g., the lower threshold at which treatment with biologics is initiated, changes in the dosing regimen) [24,25]. In the case of infliximab and etanercept, the data in this study showed that year-over-year increases (%) in use occurred shortly after biosimilar entry (13.4% increase for infliximab and 13.7% increase for etanercept).

The compound annual growth rate (CAGR) of sales volume (DDDs) indicated different growth trends for infliximab and etanercept (see Figure 1). While growth intensified for etanercept in the last few years, it decreased in the case of infliximab. This suggests a saturation of the market for infliximab, which has been subject to the competitive pressure of TNFα inhibitor therapies with a different administration profile and approval for an extended range of indications [7].

**Figure 2:**
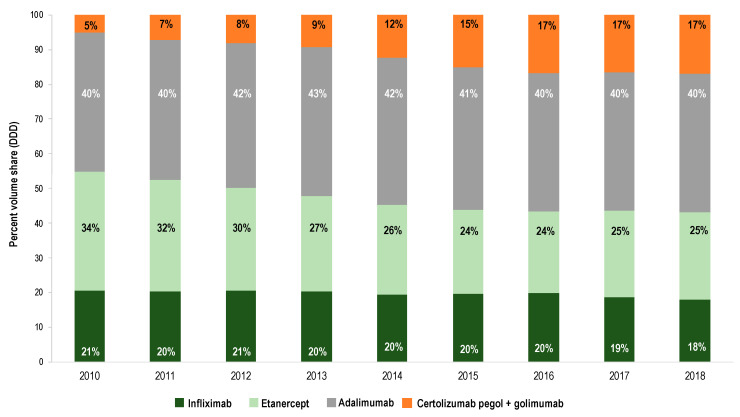
Composition of the market for TNFα inhibitors in terms of individual products from 2010 to 2018. The sales volume (DDDs) for infliximab (originator + biosimilars), etanercept (originator + biosimilars), adalimumab (originator + biosimilars), certolizumab pegol and golimumab is expressed as a share of the total volume of TNFα inhibitors.

Figure 2 shows the composition of the market for TNFα inhibitors in terms of individual products from 2010 until 2018. In 2010, the greatest volume share corresponded to adalimumab (40%), while the shares for infliximab and etanercept were 21% and 34%, respectively. The volume share for the innovative products Cimzia^®^ and Simponi^®^ amounted to 5%. During a nine-year time period, the volume share for adalimumab remained stable, while the shares for infliximab and etanercept decreased in favor of the originator therapies Cimzia^®^ and Simponi^®^. The data represented in Figure 2 indicate that the market entry of infliximab biosimilars (2015) has not induced a shift in prescribing trends from other TNFα inhibitor originator products (Enbrel^®^, Humira^®^, Cimzia^®^, Simponi^®^) towards infliximab-containing products. This observation also applies to the entry of etanercept biosimilars in 2016.

#### 2.2.2. Infliximab and Etanercept Biosimilars and Originators: Evolution in Market Shares for the German Regions

**Figure 3:**
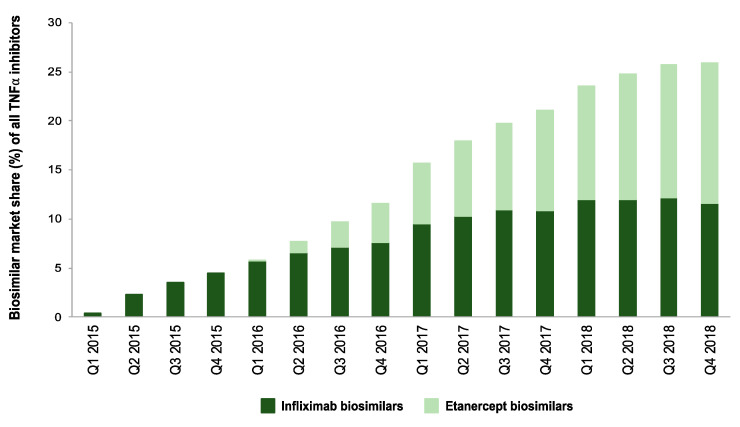
Composition of the market for TNF α inhibitors in terms of biosimilar products (2015–2018). Biosimilar market shares (%) for infliximab and etanercept are calculated in relation to the total volume of TNFα inhibitors.

At the end of 2018, the combination of biosimilar products for infliximab and etanercept represented, in terms of sales volume (DDDs), 25% of the German market for TNFα inhibitors (see Figure 3).

**Figure 4:**
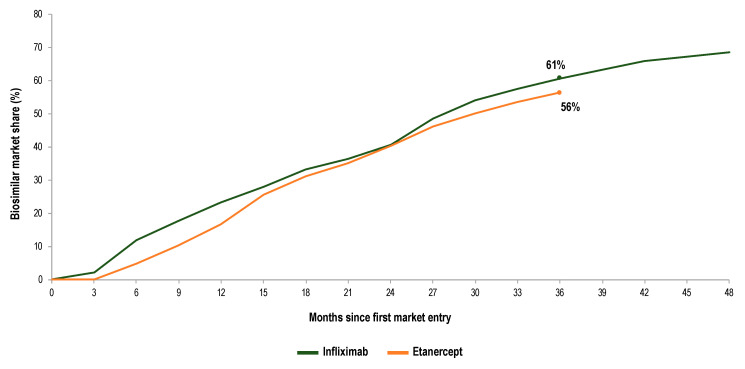
Biosimilar penetration for infliximab and etanercept in Germany over time. Biosimilar market shares (%) are calculated as volume of biosimilars over volume of biosimilars plus the originator product (DDDs).

The level of biosimilar penetration for infliximab and etanercept was comparable (56% and 61%, respectively) in Germany at the end of the third year after biosimilar market entry (see Figure 4). However, regional data on the uptake of infliximab and etanercept biosimilars (Q4 2018) pointed to a wide variation in biosimilar market shares between the 17 German PA regions (see Table 1). In the case of infliximab, the lowest biosimilar market share was observed for Brandenburg (33%), while the largest value was observed for Lower Saxony (87%). In a similar way, there was large variance of market shares for etanercept, with Brandenburg being the region with the lowest biosimilar uptake (33%) and Westphalia-Lippe being the highest (77%). The time evolution analysis of market shares showed that in general, regions with an early adoption of biosimilars (e.g., Northrhine, Westphalia-Lippe) also reached high biosimilar uptake levels (Q4 2018). Exceptions to this trend were identified (e.g., Brandenburg). While Brandenburg behaved as an early adopter of infliximab biosimilars, uptake levels at the end of 2018 were low.

**Figure 5:**
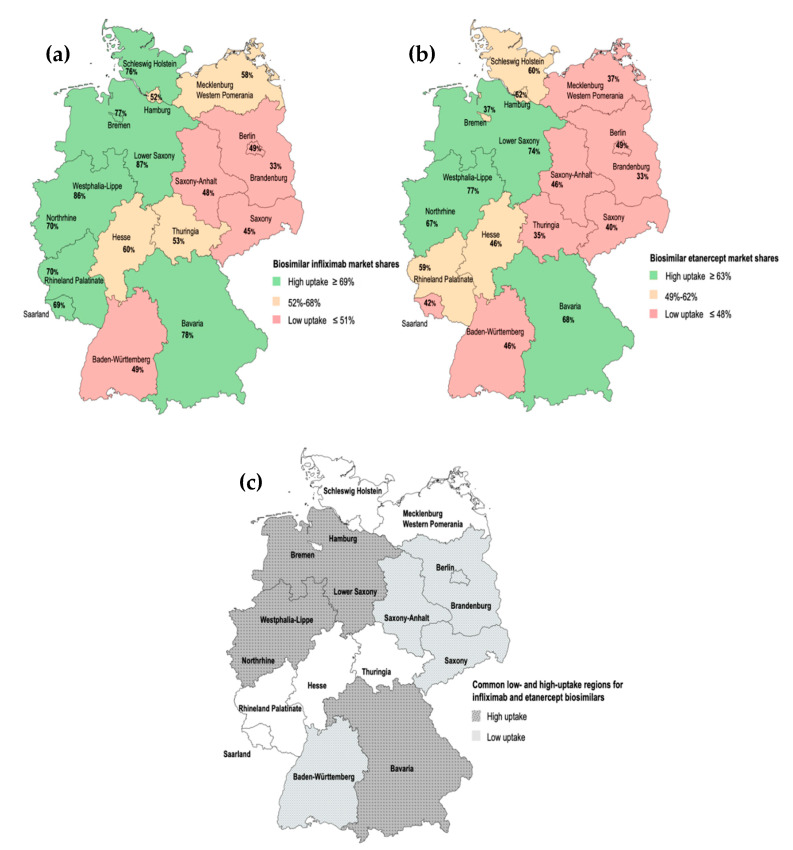
(**a**) Market shares (%) of biosimilar infliximab in Q4 2018. (**b**) Market shares (%) of biosimilar etanercept in Q4 2018. (**c**) Low- and high-uptake PA regions which are common for infliximab and etanercept biosimilars in Q4 2018. The dotted pattern refers to regions with high biosimilar uptake for both infliximab and etanercept. The crossed pattern refers to regions with low biosimilar uptake for both infliximab and etanercept. The map templates were extracted from mapchart.net.

Biosimilar uptake patterns for infliximab and etanercept were similar across Germany in Q4 2018 (see Figure 5). Indeed, regional biosimilar market shares for infliximab and etanercept were positively correlated (adjusted R^2^ = 0.64). This allowed us to identify common low- and high-biosimilar uptake regions. Saxony, Saxony-Anhalt, Brandenburg, Berlin and Baden-Württemberg showed low uptake for both infliximab and etanercept biosimilars. On the contrary, Lower Saxony, Westphalia-Lippe, Bavaria and Northrhine showed high uptake for both infliximab and etanercept biosimilars (see Figure 5c).

Figure 5 shows a predominant location of low-uptake regions within the regions formerly forming East Germany (Brandenburg, Mecklenburg Western Pomerania, Saxony, Saxony-Anhalt and Thuringia). The statistical analysis conducted (see Section 4) indicated that biosimilar market shares were significantly lower in former East Germany when compared to former West Germany. The dichotomous variable East/West location has been considered a potential co-founder in this study. In order to identify underlying predictor variables behind variable biosimilar uptake, multiple bivariate regression models were conducted to study the statistical association between a number of determinants of socio-economic welfare and regional biosimilar market shares. This is further detailed in Section 4. None of the chosen socio-economic predictor variables were found to be significantly correlated to regional biosimilar market shares.

### 2.3. The Role of Biosimilar Policies and Practices on Biosimilar Uptake: Interview Results

Physician associations regard biosimilars as a tool for economic prescribing and recommend that physicians initiate eligible patients on biosimilars and switch from the reference product to the biosimilar when possible. The view of physician associations has been generally consistent across Germany, with some discrepancies on the importance given to maintaining the prescriber’s choice over an argument of prescribing more economically. Relatively high price differences between the biosimilar and the originator product after discounting were regarded by interviewees as a driver for increased biosimilar use (see Table 2). Sickness fund representatives from the Saxony/Thuringia area signaled that physicians may prefer to prescribe discounted originators over biosimilars when price differences are small. This may explain the comparatively low biosimilar infliximab and etanercept market shares in this area and, in general, in regions formerly forming East Germany. When asked for reasons behind the lower biosimilar uptake in former East Germany regions, interviewees pointed to the past reliance of eastern Germany physicians on the strategies applied by originator companies to increase customer fidelity. This may have created stronger historical bonds between physicians and originator manufacturers that are still present today. It was also signaled that low biosimilar market shares do not necessarily reflect inefficiency regarding economic prescribing, but reliance on alternative cost-containment mechanisms.

In contrast, physician and sickness fund representatives from Westphalia-Lippe supported the prescription of biosimilars over originators, regardless of the real price difference realized after discount agreements. Here, the strategic long-term perspective relied on viewing biosimilars as a tool to reduce the increasing economic pressure that threatens the sustainability of the German Healthcare System.

#### Incentives for Increased Biosimilar Use

As stated by interviewees, certain policies/practices may affect the market penetration of biosimilars. These can differ between regions and be associated with regional variations in biosimilar market shares. Table 2 summarizes the identified drivers and facilitators for increased biosimilar use in Germany.

The establishment of biosimilar quotas was consistently identified as an important control instrument to drive biosimilar use. Interviewees indicated that the success of quotas depends on the effectiveness of the mechanisms put in place to monitor adherence to these quotas, as well as on the presence of mechanisms to sanction non-adherence. Interviewees agreed on the importance of setting out effective communication strategies to inform physicians about their accomplished prescribing rates and to facilitate biosimilar acceptance. The effectiveness of these strategies would depend to a certain extent on the robustness of the reporting capability of the regional physician associations. Interviewees also indicated that the preferential use of biosimilars over originator products has been driven by the organization of gainsharing contracts between groups of physicians and insurers. An example is the BioLike initiative, launched by the insurer company Barmer GEK for gastroenterologists and rheumatologists in different PA regions (e.g., Hamburg, Saarland, Saxony, Schleswig-Holstein, Thuringia, Westphalia-Lippe). This initiative has led to an increased use of TNFα inhibitor biosimilars and has allowed sharing the realized savings through biosimilar prescriptions between groups of prescribers and the insurer companies [27]. One of the interviewed experts signaled the positive experience of physicians with this initiative in Westphalia-Lippe.

Several German organizations, including the Drug Commission of the German Medical Association (German: Arzneimittelkommission der deutschen Ärzteschaft (AkdÄ)), the Federal Association of German Hospital Pharmacists (German: ADKA - Bundesverband Deutscher Krankenhausapotheker) and the Paul Ehrlich Institute (German: Paul Ehrlich Institut (PEI)), have published favorable statements on the safety of switching between a reference product and its corresponding biosimilars, as well as between biosimilars [28,29,30]. Interviewees mentioned that the publication of these statements may especially drive biosimilar acceptance in regions where biosimilar uptake has historically been low (e.g., Baden-Württemberg). The views of stakeholders with respect to the benefits of allowing the pharmacy-level automatic substitution of biologics (GSAV) are more divided. While physician representatives have mostly expressed doubts about the added benefit of implementing this policy, insurers have regarded it as an instrument for increased biosimilar use.

Interviewees participating in this study were also asked to identify factors primarily associated to regional variations in biosimilar uptake. Both the differing regional-level implementation of biosimilar quotas and the varying characteristics of procurement contracts appeared as important contributors. Through a comparative analysis of regional agreements for biosimilar prescription quotas, the current study showed variability in the way the national recommendations have been implemented regionally (see Table 3). Most regions have established binding biosimilar quotas. However, other regions have defined prescribing targets to be interpreted as recommendations. While biosimilar quotas for Westphalia-Lippe and Northrhine have historically been more ambitious than the national reference, Baden-Württemberg has only established non-binding recommendations for specialists known to be more familiar with TNFα inhibitor biosimilars (gastroenterologists and rheumatologists).

A certain flexibility has been allowed as well in the design and implementation of “open-house rebate contracts” established at the insurer-manufacturer level. Interviewees indicated that this may lead to intra- and inter-regional variability in biosimilar uptake. Interviewees in Westphalia-Lippe, Saxony and Thuringia reported the possibility of sickness funds to follow more or less aggressive strategies, depending on the magnitude of the pre-specified discount set as an entry requirement. Contract participants may be asked to offer the maximum level of discount possible and entry requirements may be set in a way that the differences in prices between the contract participants are minimized. These strategies may discourage the participation of originator manufacturers, leading to no discounts being negotiated for the originator. This limits the cost-savings potential that insurers could have attained through lower net prices for originator products. Therefore, insurers may adopt a less aggressive strategy where they ask for the maximum discount that the originator company is willing to provide. This strategy, although it may meet the insurer’s cost-containment objectives, results in lower than expected reductions of prices after biosimilar market entry.

## 3. Discussion

Across Europe, the level of market penetration for biosimilars has been described to be country- and product-class-specific [7,48]. In Germany, we have found similar levels of market penetration for infliximab and etanercept biosimilars at the end of 2018. However, we had expected higher biosimilar market penetration for etanercept due to the experience already gained by the market presence of infliximab biosimilars. The lower than expected market penetration of etanercept biosimilars could be partly explained by the different competition strategies followed by originator companies, which were reported to be more aggressive in the case of etanercept. Interviewees also indicated that the different administration routes for infliximab (intravenous) and etanercept (subcutaneous) may have played a role. The switch from Enbrel^®^ to etanercept biosimilars implies changes in the administration device used by patients when self-administering the drug, while this is not the case for the switch from Remicade^®^ to infliximab biosimilars.

Previous biosimilar uptake studies in Sweden [17,18] and the current study for Germany have shown that biosimilar market penetration is also region-specific and that there are wide regional variations in biosimilar market shares for TNFα inhibitors [19]. Our study of biosimilar market shares across the German regions showed common high and low uptake regions for infliximab and etanercept biosimilars. The data on market shares for adalimumab biosimilars (up to 2020) [49] indicate that regions where the uptake of infliximab and etanercept biosimilars has been high, also behaved as early adopters for adalimumab biosimilars. Therefore, we presume that biosimilar incentive policies applied regionally have had a consistent effect on the incorporation of biosimilars for the whole class of TNFα inhibitors. This observation might not be applicable to other biologic therapies (e.g., filgrastim, follitropin α) for which biosimilar uptake patterns differ from the patterns described along this study [49]. Several studies have investigated biosimilar policies implemented across Europe to qualitatively assess their impact on biosimilar uptake [50,51]. Instead, the current study examined regional variations in biosimilar uptake in order to derive practices/incentives influencing biosimilar use. Studies published by Moorkens et al. [17,18] followed this approach and were among the first to identify factors driving biosimilar use through quantitative analysis [52,53,54]. According to Moorkens et al., the absolute/relative difference in discounted price between originator and biosimilars influence decision-making regarding biosimilar use in Sweden [17]. We have not quantitatively evaluated price effects on biosimilar uptake, as information on discounted/rebated prices was not available. However, as described in the following section, we have been able to identify a set of incentive measures driving the use of infliximab and etanercept biosimilars in Germany.

### 3.1. Incentives for Increased Biosimilar Use

This study described different approaches taken by the German regions to implement a system of biosimilar prescription quotas. More active (e.g., Westphalia-Lippe) and less active (e.g., Baden-Württemberg) approaches were identified. Baden-Württemberg constituted an example of a region where the implementation of biosimilar quotas was lenient and biosimilar uptake levels were low. The role of lenient approaches on lack of adherence to biosimilar quotas has been commonly reported [27,48]. The current study, however, indicates the importance of setting instruments to support adherence with biosimilar quotas. Interviewees identified that these instruments are an effective monitoring and sanctioning system and an effective communication strategy to bridge the objectives of insurers, physician associations and individual prescribers. The capacity of regional physician associations to actively communicate with physicians and to regularly report on achieved uptake levels has been suggested as a factor driving biosimilar use in Westphalia-Lippe [55].

In Germany, the discounts realized through the establishment of “open-house rebate contracts” are confidential. The real price difference between biosimilars and the respective originator product is usually not known by prescribers. However, sickness funds are aware of the magnitude of the discounted price difference between the originator and the biosimilar alternatives. Interviewees indicated that this may define the commitment of insurers to incentivize biosimilar use over the use of discounted originator products. Based on this, the investment in educational and other resources needed to encourage biosimilar use may vary for the different sickness funds and for the different regions. Gainsharing initiatives established across Germany are an example of the active involvement of sickness funds with the promotion of biosimilars. Some of these initiatives have opted to inform participating physicians on net prices realized through discounting. It has been suggested that this approach might increase the interest of physicians on the principles of cost-effective prescribing [27]. The publication of favorable statements on the safety of switching between reference products and biosimilars is also an example of the active involvement of scientific expert committees. We hypothesize that these committees operate as opinion leaders in Germany, having an influence on prescriber’s decision-making regarding biosimilars.

Finally, the proposal to implement a policy for the automatic substitution of biologics at the pharmacy level (GSAV) has elicited conflicting views among stakeholders in healthcare [19]. We hypothesize that this measure may have a considerable impact on biosimilar uptake, potentially equalizing differences in biosimilar market shares across Germany. Further research would be needed to evaluate whether the implementation of this measure substantially changes the situation described in this study.

### 3.2. Study Limitations

The analysis of market dynamics for the class of TNFα inhibitors was based on the availability of data from ambulatory prescriptions covered by the SHI funds. The lack of information on prescriptions issued by the PHI system or at the hospital level was not expected to affect the comprehensiveness of the analysis, as most sales volume for TNFα inhibitors has been generated within the ambulatory care sector and the SHI scheme is covering 87% of Germany’s population [8,56].

We conducted a regression analysis to assess the statistical relationship between several variables chosen as predictors and the outcome variable (biosimilar market shares). We could only include descriptors of socio-economic welfare and performance indicators for the different regional healthcare systems as explanatory variables. Due to the lack of publicly available data, we could not study the association between procurement contract conditions/real differences in discounted prices between originators and biosimilars and regional biosimilar market shares. According to the view of the experts interviewed for this study, we hypothesize that these factors may better explain regional-level variability in biosimilar market shares. The availability of a limited number of observations (N = 16; we combined the data from Northrhine and Westphalia-Lippe) also conditioned the analysis: only the association between two predictor variables and market shares could be modelled simultaneously.

The qualitative analysis of interview data supplemented findings from the quantitative analysis and identified regional predictors of biosimilar uptake that could not have been easily quantified or proxied. However, it must be noted that these interviews were carried out only in nine of the 17 German PA regions. The lack of representation of every region is expected to have only a moderate impact on the generalizability of the study findings, as the interviewed regions represent > 50% of the sales volume for TNFα inhibitors in Germany.

### 3.3. Future Research

The current study provides an overview of market dynamics for the class of TNFα inhibitors in Germany and especially evaluates the evolution in sales volume for all TNF α inhibitors after the market entry of infliximab and etanercept biosimilars. To accurately evaluate the impact of biosimilar entry within the class of TNFα inhibitors, we would have needed to study the evolution in costs per molecule and per patient before and after the market launch of TNFα inhibitor biosimilars. This analysis could not be conducted due to the lack of publicly available data, but it constitutes an interesting starting point for future studies.

As part of this study, we have stressed the influence of biosimilar policies/practices for prescription and budget control on biosimilar uptake. However, the implementation success for these policies has varied across the German regions. It might be useful for future analyses to evaluate the cumulative effect of implementing multiple incentive policies/practices and to see how this effect relates to observed biosimilar market shares for the regions.

## 4. Materials and Methods

The methodology chosen for this study is based on previous studies that investigated factors influencing biosimilar uptake in Sweden [17,18]. We first conducted a literature review to describe the main characteristics of the German market for TNFα inhibitors. For reasons of international comparability, we refer to German-specific terminology identified through the literature search by using the English equivalent term. A glossary table (see Table 4) with English terms used in this manuscript and their German equivalent is provided below. Then, we examined dispensing data on sales volume and biosimilar market shares for this drug class. In order to investigate potential factors behind the variable regional uptake of infliximab and etanercept biosimilars, we relied on quantitative and qualitative analyses conducted in parallel, as detailed in the following subsections.

### 4.1. Literature Review

The main characteristics of the German healthcare system in dealing with biologics, including biosimilars, were extracted from a literature review. PubMed, Embase and Scopus were searched up to December 2019 to yield information on combined searches including the terms: policies, practices, measures, biosimilars and Germany. Studies in English and German were accepted. The website of the Federal Ministry of Justice and Consumer Protection (German: Bundesministerium der Justiz und für Verbraucherschutz (BMJV)) was accessed to retrieve relevant articles from the German Social Code Book (SGB) V [57]. Additionally, the websites of the KBV [58], the different KVs [31,32,33,34,35,36,37,38,39,40,41,42,43,44,45,46,47], and the GKV-SV were consulted [59].

### 4.2. Analysis of Dispensing Data for TNFα Inhibitors

Regional data on sales volume and uptake of TNFα inhibitor originators and biosimilars were provided by the database of the German Institute for Drug Use Evaluation (German: Deutsches Arzneiprüfungsinstitut e.V. (DAPI)). This database contains anonymous claims data of drugs prescribed and subsequently dispensed by community pharmacies at the expense of the SHI Funds. Nearly 87% of Germany’s population is insured by the SHI system [8,56]. The DAPI database covers all claims data from a representative sample of more than 80% of the community pharmacies throughout all regions. Dispensing data were linked to the database of the ABDA – Federal Union of German Associations of Pharmacists (German: ABDA—Bundesvereinigung Deutscher Apothekerverbände e.V.) containing information about the (brand) name, composition, active ingredient, strength, package size, dosage form, and route of administration of German medicinal products [60]. Defined daily doses (DDDs) [61] were calculated from dispensing data and extrapolated by regional factors to 100% of all community pharmacies, and thus 100% of the SHI insured population.

For this analysis, drug use data were examined from the first quarter (Q1) of 2010 to the last quarter (Q4) of 2018. The study of the evolution of sales volume (DDDs) for all marketed TNFα inhibitors allowed us to visualize the effect of the market entry of infliximab and etanercept biosimilars. In addition, shifts in drug utilization trends across the class of TNFα inhibitors were described following biosimilar incorporation, as well as after the market entry of the innovator therapies Cimzia^®^ and Simponi^®^. Biosimilar market shares were calculated from volume data (DDDs) and represented the volume of biosimilars over the volume of biosimilars plus the respective originator product. The evolution of biosimilar market shares for infliximab and etanercept was studied at the national level and across the 17 PA regions from the quarter in which the biosimilar entered the market (Q1 2015 for infliximab; Q1 2016 for etanercept) to the last quarter of 2018. The regional analysis of market shares allowed the identification of high- and low-biosimilar uptake regions. Uptake was considered to be high in regions where biosimilar market shares were ≥69% for infliximab and ≥63% for etanercept, and low in regions where market shares were ≤51% for infliximab and ≤48% for etanercept. (These thresholds correspond to the lower and upper third of the maximum difference in market shares observed for Q4 2018).

The predominant location of low-uptake regions within the former East Germany, i.e., Brandenburg, Mecklenburg Western Pomerania, Saxony, Saxony-Anhalt and Thuringia, led us to evaluate the statistical relationship between regional biosimilar market shares (dependent variable; N = 16) and the East/West location of the regions at a level of significance of 0.05. This univariate regression analysis was conducted with SPSS (IBM SPSS Statistics 26). Two regression models, one accounting for infliximab data and another for etanercept were built and used as a baseline for a more exhaustive statistical analysis. As the East/West location of the regions was considered to be a co-founding variable, the objective of conducting a more exhaustive analysis was to identify underlying predictor variables (socio-economic factors) behind variable biosimilar uptake. We built various bivariate regression models to examine the statistical relationship between biosimilar market shares and a set of predictors describing: (1) the variable level of socio-economic welfare across the 16 German federal states (e.g., gross domestic product (GDP) per capita, human development index) and (2) the performance of the different regional healthcare systems (e.g., number of healthcare workers employed per 1000 inhabitants, total healthcare expenditure and healthcare expenditure calculated as a share of GDP) [62]. Furthermore, we studied the correlation between regional biosimilar market shares for infliximab and etanercept to evaluate whether biosimilar uptake patterns were similar within the class of TNFα inhibitors.

### 4.3. Interviews

A total of ten semi-structured interviews (12 participants) were organized from October 2018 to February 2020 with a view to gain insight into factors potentially influencing biosimilar uptake. The conduction of interviews allowed us to complement the findings from the quantitative analysis and to investigate determinants of biosimilar uptake that could not have be evaluated quantitatively.

A selective sampling methodology was followed to achieve representation from physician associations and health insurance companies operating at the national and regional level. The interviewed representatives from these two stakeholder groups have been involved in decision-making regarding drug budget and prescription control activities and have expertise in the field of biosimilars. Participation from representatives in Baden-Württemberg, Bremen, Hamburg, Mecklenburg Western Pomerania, Lower Saxony, Saxony, Schleswig-Holstein, Thuringia and Westphalia-Lippe was achieved.

For data collection, an interview guide was drafted, validated and approved (August 2018) by the UZ/KU Leuven ethics committee (reference number: MP006423). The interview guide followed the structure of a guide previously developed by the department to study regional management of biosimilars in Sweden [17,18]. The topics were adapted for Germany through a literature search conducted as part of a master’s thesis [63]. Interview questions were organized into questions on dispensing data for TNFα inhibitors and questions on national and regional-level biosimilar policies. All interviewees received an email with an attached informed consent form and were asked for permission to record the interviews. All interviews were conducted in English via telephone calls. The recorded interviews were transcribed ad verbatim and processed using the software QSS NVivo 12. For content analysis, we built a thematic framework based on previous knowledge and findings emerging from the interviews. The results of the qualitative study were shared with the contacted interviewees for a validation exercise.

## 5. Conclusions

Variation in market penetration of TNFα inhibitor biosimilars between German regions depends on a complex interplay of multiple factors.

Experts interviewed for this study have highlighted the influence of prescription and budget control activities (organized at the regional and insurer level) on the variable uptake of infliximab and etanercept biosimilars across Germany. The use of biosimilars has been found to depend on: the regional-level implementation of biosimilar quotas, the presence of an effective monitoring and sanctioning system to regulate adherence to biosimilar quotas, the effectiveness of the communication between regional physician associations and individual prescribers, the different conditions for discount contracts established at the insurer-manufacturer level and the organization of initiatives for gainsharing. The allowance of pharmacy-level automatic substitution for biologics is expected to play a decisive role in the evolution of biosimilar consumption patterns across Germany.

## Figures and Tables

**Table 1:** Market shares (%) of biosimilar infliximab and etanercept in Germany’s 17 PA regions. Colors gradually change from red to green with increasing biosimilar market shares.

	Q1 2015	Q2 2015	Q3 2015	Q4 2015	Q1 2016	Q2 2016	Q3 2016	Q4 2016	Q1 2017	Q2 2017	Q3 2017	Q4 2017	Q1 2018	Q2 2018	Q3 2018	Q4 2018
**INFLIXIMAB**																
Lower Saxony	2%	11%	17%	24%	26%	34%	40%	48%	74%	76%	83%	84%	88%	88%	87%	87%
Westphalia-Lippe	3%	20%	25%	33%	40%	49%	57%	64%	68%	73%	77%	79%	83%	84%	86%	86%
Bavaria	2%	12%	18%	25%	30%	37%	38%	42%	55%	66%	70%	72%	75%	77%	78%	78%
Bremen	0%	3%	14%	21%	24%	10%	22%	38%	37%	48%	53%	58%	61%	71%	74%	77%
Schleswig Holstein	2%	9%	12%	13%	20%	21%	25%	27%	33%	40%	38%	47%	55%	63%	73%	76%
Rhineland Palatinate	1%	11%	17%	25%	25%	37%	41%	47%	55%	59%	60%	69%	69%	70%	62%	70%
Northrhine	1%	13%	27%	32%	40%	46%	50%	53%	57%	57%	61%	62%	63%	67%	69%	70%
Saarland	3%	24%	22%	24%	31%	33%	44%	49%	61%	57%	61%	63%	65%	67%	67%	69%
Hesse	6%	19%	28%	38%	44%	42%	43%	45%	47%	52%	51%	55%	55%	56%	58%	60%
Mecklenburg Western Pomerania	0%	0%	7%	8%	11%	12%	12%	25%	22%	28%	46%	43%	42%	43%	48%	58%
Thuringia	1%	3%	8%	15%	22%	26%	24%	23%	27%	36%	39%	50%	54%	56%	59%	53%
Hamburg	0%	1%	6%	11%	17%	14%	16%	21%	22%	25%	31%	34%	39%	43%	49%	52%
Baden-Württemberg	1%	3%	8%	12%	14%	14%	16%	21%	27%	30%	34%	41%	42%	48%	47%	49%
Berlin	0%	2%	5%	10%	15%	26%	29%	31%	36%	36%	38%	40%	43%	45%	44%	49%
Saxony-Anhalt	8%	29%	35%	31%	27%	27%	27%	25%	31%	42%	43%	46%	51%	53%	53%	48%
Saxony	0%	3%	4%	9%	10%	14%	14%	16%	18%	20%	22%	29%	33%	36%	40%	45%
Brandenburg	7%	21%	25%	25%	27%	30%	29%	31%	30%	29%	28%	29%	30%	33%	34%	33%
**ETANERCEPT**																
Westphalia-Lippe	0%	0%	0%	0%	0%	22%	38%	47%	55%	59%	62%	66%	71%	75%	76%	77%
Lower Saxony	0%	0%	0%	0%	0%	3%	9%	25%	48%	55%	61%	65%	69%	70%	73%	74%
Bavaria	0%	0%	0%	0%	0%	4%	10%	17%	34%	46%	50%	55%	61%	63%	65%	68%
Northrhine	0%	0%	0%	0%	0%	8%	14%	20%	28%	33%	39%	47%	54%	59%	63%	67%
Hamburg	0%	0%	0%	0%	0%	1%	5%	11%	17%	21%	22%	27%	40%	49%	57%	62%
Schleswig Holstein	0%	0%	0%	0%	0%	4%	7%	11%	17%	22%	28%	31%	44%	53%	57%	60%
Rhineland Palatinate	0%	0%	0%	0%	0%	5%	11%	19%	30%	35%	40%	46%	51%	53%	56%	59%
Bremen	0%	0%	0%	0%	0%	3%	8%	18%	28%	29%	37%	39%	47%	47%	54%	55%
Hesse	0%	0%	0%	0%	0%	4%	13%	15%	19%	22%	24%	28%	32%	35%	41%	50%
Saxony-Anhalt	0%	0%	0%	0%	0%	2%	5%	7%	11%	16%	21%	28%	39%	44%	43%	46%
Baden-Württemberg	0%	0%	0%	0%	0%	4%	11%	16%	23%	26%	30%	32%	37%	38%	42%	46%
Saarland	0%	0%	0%	0%	0%	4%	8%	10%	18%	19%	19%	25%	26%	34%	34%	42%
Saxony	0%	0%	0%	0%	0%	1%	3%	6%	9%	13%	17%	24%	31%	37%	39%	40%
Berlin	0%	0%	0%	0%	0%	2%	6%	11%	16%	19%	22%	26%	28%	32%	35%	37%
Mecklenburg Western Pomerania	0%	0%	0%	0%	0%	2%	4%	8%	13%	16%	18%	21%	26%	30%	36%	37%
Thuringia	0%	0%	0%	0%	0%	1%	2%	4%	5%	9%	11%	17%	23%	25%	33%	35%
Brandenburg	0%	0%	0%	0%	0%	3%	6%	10%	12%	14%	16%	19%	24%	25%	31%	33%

**Table 2:** Summary of factors identified to drive biosimilar use and facilitate biosimilar acceptance in Germany. These factors have been identified through the qualitative analysis of interview data.

Drivers of Biosimilar Use	Factors Facilitating Biosimilar Acceptance
Biosimilar prescription quotas: -Efficient monitoring -Presence of a sanctioning mechanism	Efficient communication between stakeholders -Robust reporting capability of regional physician associations
Greater cost-savings potential associated to biosimilars	
Gainsharing contracts	
Position statements/guidelines on the safety of switching	

**Table 3:** Comparative analysis of regional quota agreements for TNF⍺ inhibitors, based on information available on the websites of the 17 German PA regions [31,32,33,34,35,36,37,38,39,40,41,42,43,44,45,46,47]. Quotas were set either generally for biosimilars or more specifically for the therapeutic group or for each of the active substances within the therapeutic group. Quotas may apply to all prescribers or to specific medical specialties.

Quota Agreements: Characteristics						
Regions	Early Quota Adoption: (Before 2016)	Set Unspecifically for Biosimilars	Set for the Category of TNFα Inhibitors	Set for the Active Substance	Applied Generally to All Prescribers	Applied Differently per Specialty
Baden-Württemberg				√		√
Bavaria	√		√			√
Berlin				√	√	
Brandenburg			√			√
Bremen				√	√	
Hamburg			√			√
Hesse				√	√	
Mecklenburg Western Pomerania: (missing data)						
Lower Saxony	√			√	√	
Northrhine			√			√
Rhineland Palatinate			√			√
Saarland				√	√	
Saxony			√			√
Saxony-Anhalt		√				√
Schleswig Holstein				√	√	
Thuringia			√			√
Westphalia- Lippe	√			√		√

**Table 4:** Glossary of English/German terms and abbreviations.

English Term	German Term	German Abbreviation
Drug Commission of the German Medical Association	Arzneimittelkommission der deutschen Ärzteschaft	AkdÄ
Federal Association of Statutory Health Insurance Physicians	Kassenärztliche Bundesvereinigung	KBV
ADKA - Federal Association of German Hospital Pharmacists	ADKA - Arbeitsgemeinschaft Deutscher Krankenhaus Apotheker e.V.	-
Federal Joint Committee	Gemeinsamer Bundesausschuss	G-BA
Federal Ministry of Justice and Consumer Protection	Bundesministerium der Justiz und für Verbraucherschutz	BMJV
ABDA - Federal Union of German Associations of Pharmacists	ABDA - Bundesvereinigung Deutscher Apothekerverbände e.V.	-
German Institute for Drug Use Evaluation	Deutsches Arzneiprüfungsinstitut e.V.	DAPI
German law for more safety in the supply of pharmaceuticals	Gesetz für mehr Sicherheit in der Arzneimittelversorgung	GSAV
German federal states	Bundesländer	-
German Regional Associations of Statutory Health Insurance Accredited Physicians (also referred to in text as PA regions): **To be noted**: -This paper makes a distinction between the 16 German federal states and the 17 PA regions. Although Germany is divided into 16 federal states, the areas Northrhine and Westphalia-Lippe within the state Northrhine-Westphalia are represented by two independent PA regions. -Dispensing data have been provided/analysed per PA region and the univariate regression study has been conducted with data at the state level. This was due to limitations in data availability for the univariate regression analyses. -When referring to regions formerly forming East Germany, we include Brandenburg, Mecklenburg Western Pomerania, Saxony, Saxony-Anhalt and Thuringia, but not Berlin. This is because we do not have sub regional data to analyze uptake differences between areas formerly forming East and West Berlin.	Kassenärztliche Vereinigungen	KV
National Association of Statutory Health Insurance Funds	Gesetzliche Krankenversicherung-Spitzenverband	GKV-SV
National advisory agreement on spending targets: (also referred to in text as national-level agreements on prescription targets)	Bundesrahmenvorgaben für die Arzneimittelvereinbarungen	-
“Open-house rebate” contracts	Open-House-Rabattverträge	-
Private Health Insurance (abbreviated in text as PHI)	Private Krankenversicherung	PKV
Regional agreements on prescribing spending targets, supply and economy targets (also referred to in text as regional-level contracts to establish prescribing quotas)	Arzneimittelvereinbarungen	-
Sickness Funds (also referred to in text as insurer organizations or insurers)	Krankenkassen	-
Social Code Book V (Statutory Health Insurance)	Sozialgesetzbuch V (Gesetzliche Krankenversicherung)	SGB V
Statutory Health Insurance (abbreviated in text as SHI)	Gesetzliche Krankenversicherung	GKV

## Data Availability

The datasets generated during and/or analyzed during the current study are available from the corresponding author on reasonable request.
